# Fitness dynamics within a poplar hybrid zone: II. Impact of exotic sex on native poplars in an urban jungle

**DOI:** 10.1002/ece3.1028

**Published:** 2014-04-19

**Authors:** Amanda D Roe, Chris JK MacQuarrie, Marie-Claude Gros-Louis, J Dale Simpson, Josyanne Lamarche, Tannis Beardmore, Stacey L Thompson, Philippe Tanguay, Nathalie Isabel

**Affiliations:** 1 Natural Resources Canada, Canadian Forest ServiceQuébec, Québec, Canada; 2 Natural Resources Canada, Canadian Forest ServiceSault Ste. Marie, Ontario, Canada; 3 Natural Resources Canada, Canadian Forest ServiceFredericton, New-Brunswick, Canada; 4 Umeå University, Department of Ecology and Environmental Science, Umeå Plant Science CentreUmeå, Sweden

**Keywords:** Anthropogenic disturbance, disease susceptibility, hybridization, *Populus*, postzygotic fitness, SNP genotyping, urban–forest interface

## Abstract

Trees bearing novel or exotic gene components are poised to contribute to the bioeconomy for a variety of purposes such as bioenergy production, phytoremediation, and carbon sequestration within the forestry sector, but sustainable release of trees with novel traits in large-scale plantations requires the quantification of risks posed to native tree populations. Over the last century, exotic hybrid poplars produced through artificial crosses were planted throughout eastern Canada as ornamentals or windbreaks and these exotics provide a proxy by which to examine the fitness of exotic poplar traits within the natural environment to assess risk of exotic gene escape, establishment, and spread into native gene pools. We assessed postzygotic fitness traits of native and exotic poplars within a naturally regenerated stand in eastern Canada (Quebec City, QC). Pure natives (*P. balsamifera* and *P. deltoides* spp. *deltoides*), native hybrids (*P. deltoides* × *P. balsamifera*), and exotic hybrids (trees bearing *Populus nigra* and *P. maximowiczii* genetic components) were screened for reproductive biomass, yield, seed germination, and fungal disease susceptibility. Exotic hybrids expressed fitness traits intermediate to pure species and were not significantly different from native hybrids. They formed fully viable seed and backcrossed predominantly with *P. balsamifera*. These data show that exotic hybrids were not unfit and were capable of establishing and competing within the native stand. Future research will seek to examine the impact of exotic gene regions on associated biotic communities to fully quantify the risk exotic poplars pose to native poplar forests.

## Introduction

Introgression is the movement of genetic material from one gene pool into another through the processes of hybridization and backcrossing (Anderson 1949). The first step toward introgression is hybridization, a pervasive phenomenon in natural systems. The evolutionary consequences of hybridization and introgression are context dependent (Mallet 2005; Simberloff et al. 2013). For instance, they can lead to increased genetic diversity, novel genotypes, adaptive genetic variation, and even new hybrid species (Seehausen 2004; Arnold and Martin 2010; Abbott et al. 2013; Dittrich-Reed and Fitzpatrick 2013). However, hybridization and introgression that are the result of human-related activities are known to have effects on native populations that are perceived as negative (Allendorf et al. 2001; Potts et al. 2003; Facon et al. 2006; Schierenbeck and Ellstrand 2009; Ellstrand et al. 2010; Consuegra et al. 2011; Crispo et al. 2011; Gilman and Behm 2011; Hoban et al. 2012; Vonlanthen et al. 2012). When hybridization and introgression involve exotic species, they can lead to demographic swamping, genetic pollution, invasive hybrid lineages, or they may confer a selective advantage for individuals bearing exotic traits (Rhymer and Simberloff 1996; Ellstrand 2003, 2008; Hails and Morley 2005; Whitney and Gabler 2008; Wilkinson and Tepfer 2009; Laikre et al. 2010; Crispo et al. 2011). Conversely, there is the potential for exotic genetic material to provide an adaptive advantage to native species by increasing the genetic diversity or adaptive potential in parental species, thereby increasing their resistance to biotic or abiotic pressures (Chandler and Dunwell 2008; Zalapa et al. 2009; Hoban et al. 2012). As it is impossible and even undesirable to eliminate exotics from the landscape, it is essential that we consider a conciliatory approach to managing the flow of exotic genes by minimizing the risk, as well as accepting potential benefits, to native populations (Carroll 2011).

To determine the persistence of exotic hybrids and their long-term impact on natural systems, we need to examine the fate of exotic hybrids in natural populations. Poplar trees (*Populus* L.) are an excellent model system with which to explore these processes. Native poplars form distinct hybrid zones (Eckenwalder 1996; Vanden Broeck et al. 2005; Whitham et al. 2006; Lexer et al. 2010; Thompson et al. 2010), within which many exotic cultivars of poplar grow in close proximity to natural populations. This pure native – native hybrid – exotic hybrid system provides the opportunity for exotic genetic material to escape into the native gene pool (Meirmans et al. 2010; DiFazio et al. 2012; Talbot et al. 2012; Vanden Broeck et al. 2012). Most poplar cultivars are hybrids bearing a combination of gene regions from North American (*Populus balsamifera* L. or *P. deltoides* Marsh.), European (*Populus nigra* L.), and Asian (*P. maximowiczii* A. Henry) poplar (Eckenwalder 2001; Riemenschneider et al. 2001; Périnet 2007). We refer to these cultivars as exotic hybrids. It is important to detect exotic genetic material and quantify its spread and impact on the genetic integrity of native poplar populations. Identification of existing exotic hybrid poplars is possible using morphology, although detection has been improved through the use of molecular markers (Smulders et al. 2001; Meirmans et al. 2007; Talbot et al. 2011; Isabel et al. 2013). The development of diagnostic molecular markers has allowed us to detect the flow of exotic gene regions (including novel genomic regions) within native poplars, revealing a number of consistent patterns: (1) spontaneous hybridization occurs between native trees and exotic hybrids resulting in *F*_1_ and advanced generation hybrids bearing exotic gene regions (Smulders et al. 2008; Meirmans et al. 2010; Thompson et al. 2010; DiFazio et al. 2012; Talbot et al. 2012; Vanden Broeck et al. 2012); (2) the rate of spontaneous hybridization is highly variable (<0.5% to 72%) and dependent on population size and local pollen cloud composition (Meirmans et al. 2010; Thompson et al. 2010; DiFazio et al. 2012; Talbot et al. 2012; Leboldus et al. 2013); (3) the directionality of introgression is dependent on the parent species and is frequently asymmetrical (Thompson et al. 2010; Leboldus et al. 2013); (4) exotic hybrids can establish in natural environments via seed-mediated gene flow, with disturbed sites showing greater hybrid establishment than undisturbed sites (Thompson et al. 2010; Talbot et al. 2012).

Wilkinson et al. (2003) proposed a multistep pathway to assess the risk of exotic gene (novel genomic region) introgression in compatible native species. The impact of exotic gene regions on natives is dependent on (1) the frequency of hybridization, (2) the fertility of hybrid offspring, (3) the relative fitness of hybrid offspring and parental species, and (4) the effects on associated biotic communities. Introgression by exotic gene regions could lead to outbreeding depression, genetic incompatibilities, or breaking up of co-adapted gene complexes through recombination (Laikre et al. 2010) thereby reducing fitness in recipient populations, while increased fitness may occur from transgressive segregation or the production of adaptive genotypes (Mallet 2005; Schierenbeck and Ellstrand 2009; Keller and Taylor 2010; Hegarty 2012; Dittrich-Reed and Fitzpatrick 2013). Introgression may also have no impact on overall fitness and do little beyond increasing the genetic diversity of the recipient population. Escape of exotic hybrids only matters if they are capable of out-competing native species (Ellstrand and Schierenbeck 2000; Hails and Morley 2005; Wilkinson and Tepfer 2009). Quantifying the differences in fitness among native species, native hybrids, and exotic hybrids is therefore an important step toward predicting the risk exotic material poses to native populations (Wilkinson et al. 2003; Meirmans et al. 2009; Ghosh and Haccou 2010). The ability to predict this risk is needed to obtain approval for widespread commercial use of trees with novel genome regions (including exotic genes, transgenes, or any type of heritable genomics-derived modification) (Finstad et al. 2006). Many studies have examined hybridization and introgression in poplars (reviewed in Vanden Broeck et al. 2005), but few studies have examined the fitness of hybrid poplars (Schweitzer et al. 2002), particularly those bearing exotic components in a natural setting (DiFazio et al. 2012).

Poplars with exotic components express many traits that are commercially desirable—rapid growth, disease resistance, and abiotic stress tolerance—all of which could provide exotic individuals with a selective advantage over native individuals (Wilkinson and Tepfer 2009). In this study, we quantified the fitness of exotic poplar hybrids relative to that of native species and native hybrids to help inform the risk exotic gene regions pose to native poplar populations. Thompson et al. (2010) detected hybrid poplar trees bearing exotic genetic components at three naturally regenerated urban sites in eastern Canada, but did not detect exotic genes in sites dominated by natural forest, highlighting the unique nature of those sites. We chose to focus on one site, Base de plein-air de Sainte-Foy (BPSF), given its location and presence of pure native species, native hybrids, and exotic hybrids. In a companion paper (Roe et al. 2014), we demonstrated that native hybrid seed readily formed in *P. deltoides* and we detected adult hybrids were in the stand at BPSF. We failed to detect native hybrid seedlings, suggesting that additional barriers were selecting against hybrid seedlings, thereby contributing to the dynamics of the native hybrid zone. Herein, we contrast the patterns of hybrid formation and introgression we observed among the native components of the stand to the patterns observed when exotic poplars were incorporated into the analyses. We quantified rates of exotic hybridization and determined the realized rate of introgression of exotic genes into native populations at this urban–forest interface by genotyping reproductively mature trees, seedlings, and seeds with a panel of diagnostic markers. We then examined four postzygotic fitness traits (seed quantity, seed quality, seed germination, and disease susceptibility) that we previously used to assess the native components of the stand (Roe et al. 2014) and used them here to assess the fitness of exotic hybrids relative to other native components of the stand. Quantifying fitness traits and hybridization dynamics of exotic hybrids in a zone of natural hybridization will help predict the impacts of exotic hybrids on native populations at the urban–forest interface and quantify the risk posed by exotic genes and species to native tree populations (Ghosh and Haccou 2010; Ghosh et al. 2012).

## Methods

### Study site and system

**Figure 1:**
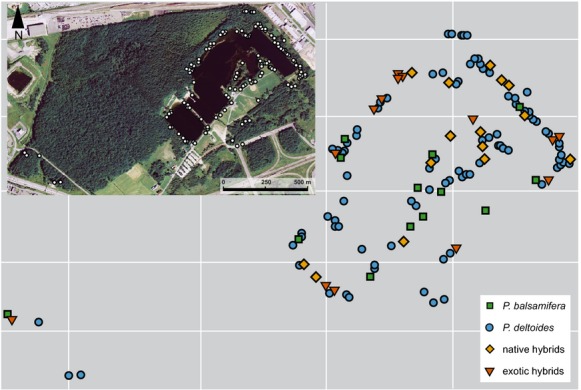
Base de plein-air de Sainte-Foy collection site in summer 2009 with the location and genotype class for each adult individual.

The BPSF is a 136 hectare recreational park that consists of a central artificial lake surrounded by mixed deciduous forest and managed grassland (Fig. 1). Vegetation at the site was allowed to naturally regenerate following gravel extraction approximately 60 years ago. The location and colonization history of BPSF have created a complex stand composed of pure native species, native hybrids, and exotic hybrids (Thompson et al. 2010). Native poplars include *P. balsamifera*, *P. deltoides,* and their natural hybrids (Roe et al. 2014). Previous surveys have detected exotic hybrids, containing *P. nigra* and *P. maximowiczii* alleles at BPSF (Thompson et al. 2010; this study). The native poplars (*P. balsamifera* and *P. deltoides*) belong to sections Tacamahaca and Aigeiros, respectively. Exotic *P. maximowiczii* belongs to section Tacamahaca, same as *P. balsamifera*, while the taxonomic classification of exotic *P. nigra* is ambiguous (Hamzeh and Dayanandan 2004).

### Sampling

Adult trees, seeds, and newly established seedlings were sampled throughout BPSF (Fig. 1). A representative number of adult trees were selected to characterize the genetic diversity at BPSF and sampled as described in Roe et al. (2014). We recorded tree location, gender, age, diameter at breast height (DBH), and height so that exotic trees could be compared with the native trees in the stand.

### Genotyping

#### DNA extraction and genotyping

Samples were prepared, extracted, and genotyped as in Roe et al. (2014). The complete SNP panel is shown in Table S1. A complete SNP data file has been deposited in the Dryad data repository (http://www.datadryad.org — http://doi:10.5061/dryad.6vk6f).

#### Classification

All trees, seedlings, and seeds were assigned to one of four genotype classes: pure *P. balsamifera* (B), pure *P. deltoides* (D), native hybrid (D × B – hybrids with only *P. balsamifera* and *P. deltoides* alleles), or exotic hybrid (X – pure *P. nigra*, pure *P. maximowiczii*, or hybrids with native alleles and *P. nigra* or *P. maximowiczii* alleles). Assignments were performed as in Roe et al. (2014), first manually by two independent observers (M Lamothe & AD Roe) and then complemented with a Bayesian clustering algorithm (Pritchard et al. 2000). To assist the structure clustering algorithm, we included an additional 234 pure reference individuals (120 B, 40 D, 22 *P. maximowiczii*, 52 *P. nigra*) to act as an a priori “learning population” (Pritchard et al. 2000). Assignment of individual trees to one of the four genotype classes (B, D, D × B, X) was determined using admixture proportions (Q-values) from structure and thresholds established from pure reference populations as in Roe et al. (2014, Table S1) and illustrated in Figure S1.

### Fitness traits

We assessed the same fitness traits for the exotic trees (X) as previously estimated for the native tree classes (B, D, D × B), which includes phenology, hybridization rate, reproductive biomass, reproductive yield, germination, and disease susceptibility (Roe et al. 2014).

### Statistical analyses

Data were analyzed using linear mixed-effect and general linear models as in Roe et al. (2014). All analyses were carried out in the R statistical computing language (R Development Core Team 2012). Supplemental files for our R analysis code are available (Data S1, http://doi:10.5061/dryad.6vk6f).

## Results

### Genotyping and classification

Two hundred and eighteen poplar samples were classified as exotics (12 adults, 202 seeds, 8 seedlings) as they contained diagnostic alleles for one of both of *P. nigra* and *P. maximowiczii*. The native samples were classified as: B (*n* = 213), D (*n* = 339), and D × B (*n* = 344) (Table 1) (Roe et al. 2014). We excluded samples with >10 missing SNP loci. We typed all exotic hybrid trees for the *trnL* intron in the maternally inherited chloroplastic DNA to determine the maternal lineage of each individual. The majority of exotic adult hybrids (8/12) had a D cpDNA lineage, although one exotic hybrid had a B maternal lineage and three exotic hybrids had a *P. maximowiczii* maternal lineage, while all adult native hybrids (*n* = 15) had a D cpDNA lineage (Roe et al. 2014).

**Table 1:** Genotype tree classification of the final data set of samples from Base de plein-air de Sainte-Foy. 1A: Numbers of adults, seedlings, and seeds of *P. balsamifera* (B); *P. deltoides* (D); hybrids with only B and D alleles (native); and hybrids containing *P. nigra* (N); and/or *P. maximowiczii* (M) alleles (exotic) as determined by consensus between manual and Q-value assignments. 1B: Putative fathers as determined by haplotype subtraction, except where father genotype could not be reconstructed with certainty (indicated by ?)

A. Genotypic classification					
	*n*	B	D	Native	Exotic
Adults	138	13	97	15	12
♂	58	5	41	8	4
**♀**	80	8	57	7	8
Seedlings	81	62	11	0	8
Seeds	900	138	231	329	202

B. Summary of half sibling progeny						Putative father												
♀(*n*)	Seed					Native			Exotic						Uncertain			
			
	*n*	B	D	Native	Exotic	B	D	DB	N	BN	MB	MBN	DBN	DN	B?	D?	BN?	DN?
*P. balsamifera* (3)	143	138	0	1	4	138		1			2	1	1					
*P. deltoides* (4)	234	0	231	3	0	3	231											
Native hybrids (7)	332	0	0	314 ( 11^1^)	7	254	60	11	5		1			1				
Exotic hybrids (4)	191	0	0	0	187 (1^1^,1^2^,2^3^)	81		5		8	1	1			90	1	3	1

1 Typed as pure but from native hybrid mother.

2 Typed as native hybrid but from exotic hybrid mother.

3 Typed as pure but from exotic mother.

**Figure 2:**
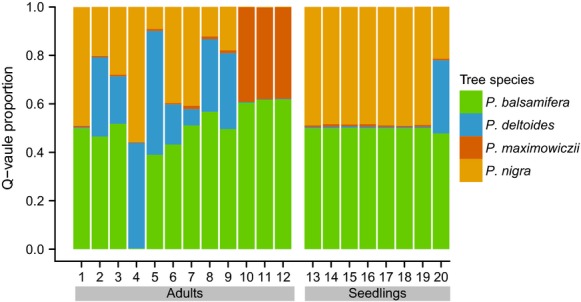
Proportions of admixture for each adult and seedling individual identified with exotic alleles from *P. maximowiczii* or *P. nigra*. Admixture proportions were based on Bayesian clustering of 36 SNP panel for *K* = 4 genomic clusters.

Exotic hybrid genotypes were complex (Fig. 2). The manual classification and Q-value assignments of these samples agreed, but the assignment methods disagreed in the classification of some exotic hybrid seeds (Table 1, Table S2). For ten seeds obtained from exotic hybrid mothers, no exotic alleles were detected, which we attribute to mistyping or segregation. An additional four seeds each contained only a single exotic allele, which the Q-value threshold method failed to detect given the conservative assignment threshold we used.

### Stand characteristics

Exotic hybrids comprised 8.7% of the sampled adult tree population at BPSF (Table 1, Fig. 1). This is similar to the observed amount of B (9.4%) and D × B (10.9%) genotypes in the stand, with the remaining 70.0% of the sampled trees having a D genotype (Roe et al. 2014). There was a significant effect of genotype class on tree height (df = 3; deviance = 585.39; residual df = 136; residual deviance = 1838.6; F* = 14.434; *Χ*^*2*^ ≤ 0.001) and DBH (df = 3; deviance = 454829; residual df = 137; residual deviance = 2349234; F* = 8.8414; *Χ*^*2*^ ≤ 0.001) (Table 2; Fig. S2). Exotic hybrids were significantly taller (*P* < 0.001) than B, but did not have greater DBH (*P* = 0.095), and were not significantly different than D or D × B (Table 2). Exotics ranged in age from ∼20 years old to 60 years old (average age 34 years ± 10; Table S3). The proportion of seedlings with exotic components was the same as the adult population (9.9%, Table 1).

**Table 2:** Stand and fitness traits measured in the complete data set for the exotic hybrids, native hybrids, *P. balsamifera*, and *P. deltoides* at BPSF. For each trait, we show the number of individual trees sampled (*n*), mean (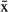), and standard deviation (SD). Stand-level traits were diameter at breast height (DBH, in millimeters) and height (in meters). Reproductive biomass traits measured were (in order): total biomass, total seed biomass, 100 seed count biomass, capsule biomass, stem biomass, and cotton biomass (in grams). Reproductive yield was measured in terms of the number of capsules per catkin, seeds per capsule, and total number of seeds per catkin. Viability was measured in terms of percent normal germination and percent abnormal germination, and due to low sampling of exotic hybrids, we only show 2009 data. Controlled fungal inoculations quantified the number of uredia/cm^2^ for three fungal species: *Melampsora larici-populina* (*Mlp*), *M. medusae* f.sp. d*eltoidae* (*Mmd*), and *M. occidentalis* (*Mo*). When significant differences were detected (Files S6–S9), post-hoc tests (Tukey's Honestly Significant Difference) were used to identify the differences between genotype classes. Different letters in the post-hoc column indicate significantly different means

Trait	Tree class															
	
	Exotic hybrid			Native hybrid			*P. balsamifera*			*P. deltoides*			Post hoc			
				
	*n*	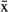	SD (±)	*n*	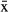	SD (±)	*n*	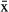	SD (±)	*n*	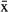	SD (±)	X	N	B	D
Stand																
DBH	14	424.00	99.61	14	412.86	99.28	14	310.43	74.16	99	490.61	143.59	ab	ab	b	a
Height	12	20.92	3.27	14	20.09	2.41	14	15.41	2.87	100	22.19	3.94	a	a	b	a
Reproductive biomass																
Total^1^	3	1.27	0.39	6	0.90	0.38	6	1.74	0.52	6	1.31	0.45	ab	b	a	ab
Seed^1^	3	0.13	0.10	6	0.10	0.059	6	0.24	0.12	6	0.22	0.098	ab	b	a	ab
100-seed	3	0.042	0.011	6	0.035	0.0081	6	0.37	0.0084	6	0.052	0.015	ab	b	ab	a
Capsule	3	0.59	0.18	6	0.34	0.12	6	0.76	0.21	6	0.52	0.19	ab	b	a	b
Stem	3	0.14	0.48	6	0.091	0.038	6	0.15	0.059	6	0.076	0.020	ab	b	a	b
Cotton	3	0.44	0.18	6	0.37	0.20	6	0.58	0.17	6	0.49	0.21	–	–	–	–
Reproductive yield																
Capsules/catkin	3	49.95	12.48	6	34.53	10.43	6	59.51	9.78	6	21.37	5.05	a	b	a	c
Seeds/capsule^1^	3	6.97	7.30	6	9.03	6.28	6	12.34	5.35	6	20.75	8.44	ab	a	ac	a
Seeds/catkin	3	305.07	261.77	6	320.66	251.35	6	656.15	330.13	6	442.08	193.39	–	–	–	–
Germination (2009)																
Germination	8	90.23	8.26	15	81.03	16.22	12	93.94	14.03	34	55.17	28.64	ab	b	ab	c
Abnormality	8	3.04	2.84	15	4.76	5.30	12	1.42	3.38	34	19.10	14.80	a	a	b	c
Fungal inoculation																
*Mlp*	6	1.01	1.33	7	0.70	0.78	3	2.35	1.87	8	0.053	0.17	a	a	b	a
*Mmd*	6	1.37	1.20	7	1.55	1.35	3	1.94	1.37	8	0.28	0.47	a	a	b	a
*Mo*	6	0.46	0.61	7	0.83	1.48	5	2.25	1.55	8	0.00	0.00	ab	b	a	ab

1 Significance differs in partial data set.

### Phenology

**Figure 3:**
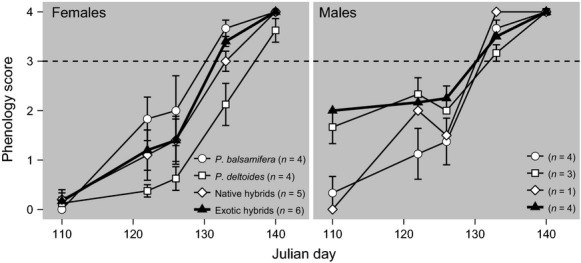
Flowering phenology of male and female *P. balsamifera*, *P. deltoides*, native hybrids, and exotic hybrids (in black). Dashed lines indicate phenological stage for pollen shedding and female receptivity.

The four genotype classes showed similar phenological timing (Fig. 3). Timing of reproduction in exotics coincided with native components of the stand, with the exception of D females which were delayed relative to the other genotype classes. Also, male (*n* = 4) exotic hybrids flowered sooner than female exotic hybrids (*n* = 6) during the early part of the season (before calendar day 130), but this asynchrony resolved itself such that both male and female flowers experienced synchronous dehiscence and receptivity later in the season (after day 130).

### Spontaneous hybridization rate and putative paternal contribution

We calculated the rates of native and exotic spontaneous hybridization rate for B, D, and D × B. We were unable to estimate hybridization rates for exotic hybrids because they are already complex mixtures of native and exotic alleles. For D × B, the hybridization rate was calculated based only on data from seeds bearing exotic components. Overall*,* B females had the highest spontaneous hybridization rate (3.50%), compared with native hybrids (2.11% – exotics only) and D (1.28%). Hybrid seeds produced by B were sired primarily by exotic males (4/5 seeds), while all hybrid seeds of genotype D were sired by genotype B fathers (Table 1, Table S2).

### Per-species rate of gene flow

In B, the majority of gene flow was intraspecific (98.30%). The contribution of D was negligible (0.52%), while the exotic contribution was higher (1.19%). In D, the majority of gene flow was also intraspecific (99.04%). The B contribution was small 0.96%, and the remaining gene flow was intraspecific as there was no exotic contribution to the seed gene pool. Among D × B, the exotic allele contribution was higher than in the pure populations (1.66%).

### Fitness

We measured reproductive biomass, yield, seed germination, and disease susceptibility among exotic hybrids and native trees within the stand at BPSF. Here, we focus on the differences between exotics and the native trees in the stand. Like in the previous analysis (Roe et al. 2014), the sampling was partially replicated among the subjects (trees) for the biomass and yield traits (Table S4). As before, we detected a weak effect of sample year on the data (Data S1), so we adopted the same approach and analyzed biomass and yield twice, once with the full data set treating all samples as independent, and again on a partial data set. The results from both data sets showed the same results, except where indicated. A summary of our results is presented herein (Table 2), for detailed results see the supplemental files (Tables S5, S6). As mentioned previously (Roe et al. 2014), our low sample sizes limit us in our ability to assess variability among fitness traits, so these values will be subject to some biases.

#### Biomass

All analyses showed a significant effect of either genotype class or year on measures of reproductive biomass (Table 2; Table S5; Fig. S3). Genotype class had a significant effect on total weight, capsule weight, hundred seed weight, and stem weight; year had a significant effect on cotton weight. The same result was seen in the analysis of the partial data set for capsule weight, hundred seed weight, and stem weight, but no effect of either factor was detected in the partial data set for total weight, cotton weight, and seed weight (Table S5). Among the genotype classes, we only observed significant differences between the native genotype classes (B, D, D × B – Table 2). The exotic hybrids were intermediate in all biomass measurements and did not differ from any of the native genotype classes.

#### Yield

We identified significant differences in reproductive yield among each genotype class (Tables 2 and S4; Fig. S3). Exotic hybrids had similar numbers of capsule per catkin to B, and significantly more than D (full, *P* < 0.001; partial, *P* < 0.001) and D × B (full, *P* = 0.01; partial, *P* = 0.01). Exotic hybrids also had the lowest number of seeds per capsule, but were only significantly different from *P. deltoides* (full, *P* < 0.001; partial, *P* = 0.01) (Table S6). Although there were significant differences in capsules per catkin and seeds per capsule, the total number of seeds per catkin did not significantly differ between any genotype class. The factors influencing yield were not consistent among the three measures, and year had no effect in any of the analyses (Table S6).

#### Germination

Exotic hybrids showed similar levels of germination success to B and D × B, and had significantly better germination than D in 2009 (*P* = 0.05) (Table 2, Table S7; Fig. S4). Exotic hybrids also had similar numbers of abnormal germinants as native hybrids, which was significantly more than B (*P* = 0.01), but fewer than D (*P* = 0.003) (Table 2, Table S7; Fig. S4). Genotype class was the only factor with a significant influence on both measures of seed germination. Models that contained only TSW were not a better fit to the data than a null model that only fit an intercept. The addition of genotype class to the model produced a significantly better fit (Table S7). The distribution of sampling among years and genotype class was not equal (Data S1), and some trees were sampled in multiple years. Despite the repeated sampling of some trees, the data for the exotic hybrids were too sparse to allow us to test the effect of both year and genotype class in the same analysis. Therefore, we tested the effect of genotype class and 1000 seed weight (TSW) on germination using just the seed collected in 2009.

#### Disease susceptibility

Genotype class had a significant effect on the number of fungal uredia in all three experiments (Table 2, Table S8). Exotic hybrids showed intermediate levels of fungal susceptibility relative to B and D, similar to D × B. Susceptibility among exotic hybrids was significantly less than B for Mlp and Mmd, but was not significantly different from other native genotype classes. Class B, D × B and X showed greater susceptibility to Mlp and Mmd than D (Fig. S5). Exotics were not significantly different than other native genotype classes for Mo susceptibility. For all three fungal species, the model containing genotype class was a better fit than a null model containing only random effects (Table S8). The raw data for these experiments contained many zero values (no uredia grew on the leaves), and the data were overdispersed (Data S1). These two issues were only partially corrected by fitting a log-Poisson model; therefore, the parameter estimates derived from these models may be overly optimistic, and the true influence of genotype class on fungal growth may be overestimated. These results should be interpreted with caution.

Field surveys of *Melampsora* rust incidence showed similar patterns to those observed in the controlled inoculations (Table S7). *Septoria* leaf spot was observed on eight of nine exotic hybrids, similar to the patterns observed among native trees (Table S9).

## Discussion

We characterized the fitness of exotics within a native hybrid zone, which allowed us to compare and contrast exotic hybridization and introgression to the same processes occurring among native poplars (Roe et al. 2014). We focused on a naturally regenerated stand with a known colonization history comprised of native poplars, hybrids, and poplars bearing exotic gene regions. This site provided a unique opportunity to examine the fitness of exotic trees over time relative to native trees. We documented spontaneous hybridization between native species and exotic hybrids and demonstrated that the fitness of exotic hybrids was intermediate to pure native species and equal to that of native hybrids. Gene flow was asymmetric, with exotic alleles predominantly introgressing into *P. balsamifera*, while hybrid formation among natives occurs primarily with female *P. deltoides* (Roe et al. 2014). As well, exotic hybrid seedlings were detected, contrasting with the absence of native hybrid seedlings within the stand. Our results show that trees with exotic components can establish, survive, and reproduce within this small urban population, potentially leading to the spread of exotic genetic material into larger native populations.

Previous surveys of introgression among native poplars across an eastern Canadian hybrid zone revealed that 2.4% of trees had hybridized with exotic poplars (*P. nigra* and *P. maximowiczii*) (Thompson et al. 2010). Exotic hybrids were restricted to disturbed urban areas and were not found in natural forests. For over a century, exotic poplar cultivars have been planted as ornamentals and windbreaks throughout North America (Richardson et al. 2007). More recently, poplar cultivars are being used for bioenergy production, carbon sequestration, and phytoremediation (Doty et al. 2007; Richardson et al. 2007; Hinchee et al. 2009; Harfouche et al. 2011). Human activities, especially in urban areas, have brought these exotics into contact with native poplar populations, thereby providing opportunities for exotic genes to escape into native gene pools (Dodet and Collet 2012). To assess the potential of exotic genes to ‘escape’, the fitness of exotic hybrids and the directionality of introgression must be quantified (Potts et al. 2003; Wilkinson et al. 2003; Wilkinson and Tepfer 2009), particularly at the urban–forest interface (Borgmann and Rodewald 2005; Thomas and Moloney 2012). Our results show that poplars bearing exotic components produce viable offspring, as both pollen donors and pollen recipients (Table 1; Table S2). Exotic poplar hybrids were not universally unfit based on our measured postzygotic fitness traits (Arnold and Hodges 1995), although fitness was variable among individuals (Table 2; Figs. S3–S5). Reproductive fitness and disease susceptibility of exotic hybrids were not significantly different from native hybrids and were intermediate to pure native species (Table 2; Figs. S3–S5).

Realized risk of exotic introgression is dependent on survival, germination, vigor, and establishment of hybrids bearing exotic components (Wilkinson et al. 2003). Exotic hybrid seedlings were competitive and viable as they comprised 10% of the seedling population, and their continued presence in the adult cohort demonstrates long-term survival and vigor. The age distribution of exotics indicates that exotic hybrids have been colonizing, competing, and reproducing in BPSF for at least 60 years in the presence of native species. The presence of exotic hybrids producing viable seed over long periods is significant. Seed-mediated gene flow is often underappreciated and may result in a greater impact than pollen-mediated gene flow rates (Chandler and Dunwell 2008; Jhala et al. 2011; Wilkinson 2011; Talbot et al. 2012).

We can apply our findings toward assessing the risk of gene flow between native and exotic species. First, exotic hybrids are often bred for traits which may provide a fitness advantage over native species (e.g., rapid growth, disease resistance, abiotic tolerance). Second, exotic hybrids growing in urban or horticultural settings are not subject to the same selective pressures as trees growing in natural stands. The proximity of BPSF to urban populations of exotic poplar cultivars may have exposed this area to high propagule pressure (Simberloff 2009; Consuegra et al. 2011) and provided a long-term source of exotic material that could establish and introgress when conditions were favorable. However, we found that the fitness of exotic hybrids was highly variable, which may be linked to underlying genetic variation (Martinsen et al. 2001; Schweitzer et al. 2002) and environmental variability (Campbell and Waser 2007). Therefore, we argue that the persistence of exotic hybrids in the landscape and the spread of genes into native populations are determined by interactions between genetic and environmental factors. These factors should be examined in greater detail to better quantify the impact of exotics on native populations.

At BPSF, exotic poplar hybrids established, survived, and produced viable offspring, and were as fit as native poplar in the same stand. This finding gives strong support to the hypothesis that exotic gene regions could spread into the native gene pool. The rate and directionality of introgression will determine the direction of evolutionary change (Petit 2004), an important consideration when exotic genetic material is introgressing into native populations (Ellstrand 2008). Our results suggest that *P. balsamifera* was more permeable to exotic gene introgression than *P. deltoides*. This was consistent with results from other hybrid zones where members of the section Tacamahaca were also more permeable to interspecific gene flow than members from other sections (e.g., section Aeigeros) (Keim et al. 1989; Stettler et al. 1996; Floate 2004), although the persistence of exotic genes within native populations will be dependent on selection. Differences in hybridization rate and directionality are dependent on the strength of postzygotic barriers, such as genetic incompatibilities, that completely or selectively prevent the formation of hybrid crosses (Zsuffa et al. 1999; Riemenschneider et al. 2001; Tiffin et al. 2001; Vanden Broeck et al. 2005; Lexer et al. 2010; Macaya-Sanz et al. 2011). Postzygotic barriers in exotic poplar hybrids may also be weaker than those in native species. The limiting step in introgression is often the first backcross of *F*_1_ hybrids to pure parental species (Rieseberg and Carney 1998; Arnold et al. 1999). However, many poplar cultivars are already complex hybrids bearing both exotic and native genes (Eckenwalder 2001; Riemenschneider et al. 2001); therefore, hybrid cultivars may be more capable of backcrossing with native species. Despite extensive study of hybridization within poplars, the pre- and postzygotic barriers controlling hybrid formation are still poorly understood and require further investigation.

We explored the impact of postzygotic fitness on the realized risk of exotic gene introgression. In addition to these intrinsic traits, introgression of exotic genes into native populations can be affected by stochastic processes (Ghosh and Haccou 2010) such as the strength of selection, population size, population structure, and population dynamics, all of which can alter the rate of spread and fixation of exotic gene regions within recipient populations (Levin et al. 1996; Lepais et al. 2009; Meirmans et al. 2009; Fitzpatrick et al. 2010; Ghosh and Haccou 2010; Laikre et al. 2010; Field et al. 2011; Ghosh et al. 2012). The frequency of introgression may also be important in the persistence of introgressed regions and affects the likelihood that they will become fixed (Ghosh et al. 2012), such that even rare hybridization and introgression events can alter the evolutionary trajectory of a population (Burke and Arnold 2001). Exotic introgression, coupled with a fitness advantage, is a potent force for evolutionary change. When advantageous exotic genes introgress into native populations, they may replace native alleles, pollute breeding material, cause outbreeding depression, act as a bridge to exotic pests, lead to weediness, and impact associated biotic communities (Rhymer and Simberloff 1996; Whitham et al. 1996, 2006; Ellstrand and Schierenbeck 2000; Abbott et al. 2003; Ellstrand 2003). Conversely, advantageous alleles may provide a source of adaptive variation, relieve inbreeding depression, and produce novel genotypes (Rieseberg and Carney 1998; Barton 2001; Abbott et al. 2003; Ellstrand 2003; Taylor et al. 2009) that could help native populations overcome changes in climate or develop resistance to disease (Chandler and Dunwell 2008). Furthermore, populations of exotic hybrids may even gain adaptive variation via introgression from their wild relatives (Petit 2004; Brown et al. 2009). This process has been documented in a number of domesticated crops, such as apple (Cornille et al. 2012), olive (Kaniewski et al. 2012), almond (Delplancke et al. 2012), and grapevine (Myles et al. 2011). Native populations are often better adapted to local conditions, and introgression of native gene regions can confer this adaptive advantage to exotic individuals leading to the creation of locally adapted domestic cultivars. Ultimately, hybrid fitness and the extent of introgression will be controlled by multiple interacting factors, and additional work is needed to assess their relative influence on hybrid fitness and exotic gene flow.

Production of exotic hybrids and human encroachment on native forests is ever increasing, providing opportunities for exotic genes to introgress into native tree populations. Broader surveys of poplar hybridization in eastern Canada found that establishment of poplars bearing exotic gene regions was limited to areas of human-mediated disturbance (Thompson et al. 2010) and there is little evidence of exotics displacing native North American species in natural environments (US Environmental Protection Agency 1999; Talbot et al. 2012; but see Vanden Broeck et al. 2005; Smulders et al. 2008). Land use patterns and propagule pressure impact the ability of exotics to invade and establish in natural ecosystems (Foster et al. 2003; Vilà and Ibáñez 2011; Hoban et al. 2012), and the encroachment of urban areas on natural forests increases disturbance and provides sources of exotic material (Borgmann and Rodewald 2005; Vidra and Shear 2008). The risk of exotic gene introgression at this urban–forest interface should be quantified to establish the overall risk that urban encroachment poses to [the genetic integrity of?] native forests. It would be valuable to examine the factors that impact the survival, establishment, and persistence of poplars bearing exotic genes with a common garden framework that could tease apart the extrinsic and intrinsic factors controlling exotic poplar fitness. We must understand the mechanisms controlling exotic gene introgression in order to maintain the genetic integrity of native populations. Quantifying the impact of exotic admixture on postzygotic fitness is one step toward predicting exotic gene introgression and developing an effective management framework to monitor and mitigate the effects of these exotic trees (Wilkinson et al. 2003).

## Supporting Information

**Figure S1.** Schematic demonstration of the thresholds used to assign the four genotype classes: pure *P. balsamifera* (B), pure *P. deltoides* (D), native hybrids (D × B), and exotic hybrids (X). Reference populations were used as an a priori learning population to develop threshold levels to assign individuals from BPSF. Individuals above the upper threshold were considered pure. If Q-values fell below the lower threshold, then alleles for that species were absent within an individual. Genotype classes were based on Q-values and CI intervals relative to the 0.5 threshold (i.e., *F*_1_) and the lower threshold (backcrosses or trihybrids).

**Figure S2**. Box plot of adult tree diameter at breast height (DBH) and height for four genotype classes: pure *P. balsamifera* (B), pure *P. deltoides* (D), native hybrids (D × B), and exotic hybrids (X). Post-hoc tests (Tukey contrasts) were used to identify differences among genotype class means.

**Figure S3**. Box plot of adult tree reproductive biomass and yield for four genotype classes: pure *P. balsamifera* (B), pure *P. deltoides* (D), native hybrids (D × B), and exotic hybrids (X). Reproductive biomass measures include: total biomass, seed biomass, 100-seed biomass, capsule biomass, stem biomass, cotton biomass. Reproductive yield measures: number of capsules per catkin, seeds per capsule, and seeds per catkin. Post-hoc tests (Tukey contrasts) were used to identify differences among genotype class means.

**Figure S4**. Box plot of adult tree seed viability for four genotype classes: pure *P. balsamifera* (B), pure *P. deltoides* (D), native hybrids (D × B), and exotic hybrids (X). Seed viability was assessed based on percent germination and percent abnormal germinants. Post-hoc tests (Tukey contrasts) were used to identify differences among genotype class means.

**Figure S5**. Box plot of adult tree disease susceptibility for four genotype classes: pure *P. balsamifera* (B), pure *P. deltoides* (D), native hybrids (D × B), and exotic hybrids (X). Disease susceptibility was assessed based on the number of uredia per cm^2^ for using controlled inoculations of three *Melampsora* species. Post-hoc tests (Tukey contrasts) were used to identify differences among genotype class means.

**Data S1**. R code for statistical analyses.

**Table S1**. Diagnostic SNP markers for native and exotic poplar species at Base de plein-air de Sainte-Foy (Table adapted from Isabel et al. 2013). Major allele frequency is 1.0 unless indicated otherwise. Gray cells indicate the potential presence of a partial null allele. *Populus trichocarpa Nisqually-1* was used as a reference sequence.

**Table S2**. Summary of half-sibling progeny from pure, hybrid, and exotic hybrid mothers. Putative fathers are inferred by haplotype substraction from maternal and seed genotypes (*P. balsamifera* = B, *P. deltoides* = D, *P. nigra* = N, *P. maximowiczii* = M; ? indicates that the father genotype could not be completely reconstructed with confidence).

**Table S3**. Distribution of age classes estimated for reproductively mature *P. balsamifera*, *P. deltoides*, and native hybrids at Base de plein-air de Sainte-Foy.

**Table S4.** Number of poplar trees measured for reproductive fitness and disease resistance traits: reproductive biomass, reproductive yield, seed viability, fungal disease susceptibility. Fungal disease was measure for three *Melampsora* species*: M. larici-populina* (*Mlp*), *M. medusae* f.sp. *deltoidea* (*Mmd*), and *M. occidentalis* (*Mo*).Trees were grouped into four genotypic classes: pure *P. balsamifera* (B), pure *P. deltoides* (D), native hybrids (D × B), and exotic hybrids (X). Numbers in brackets indicate the number of trees sampled more than once.

**Table S5**. Linear mixed-effects model analysis summary for six biomass measurments taken on catkins from trees in *P. balsamifera* (B), *P. deltoides* (D), native hybrids (D × B), and exotic hybrids (X). A: Full data set; B: Partial data set of once sampled trees. Post-hoc tests (Tukey contrasts) were used to identify differences among genotype class means.

**Table S6**. Linear mixed-effects model analysis summary for the reproductive yield of catkins sampled in *P. balsamifera* (B), *P. deltoides* (D), native hybrids (D × B), and exotic hybrids (X). **7A:** Full data set; **7B:** Partial data set. Post-hoc tests (Tukey contrasts) were used to identify differences among tree class means when a significant fixed effect was detected.

**Table S7.** General linear model analysis summary for germination of seed from trees in four genotype classes: pure *P. balsamifera* (B), *P. deltoides* (D), native hybrids (D × B), and exotic hybrids (X). Tukey's Honestly Significant Difference test was used to identify differences among genotype class means when a significant effect was detected (Table 2).

**Table S8**. Linear mixed-effects model analysis summary for infection severity (number of uredia) of poplar rust on trees in four genotypic classes: pure *P. balsamifera*, pure *P. deltoides*, native hybrids, and exotic hybrids. Results are based on controlled inoculations of *Melampsora larici-populina* (*Mlp*), *M. medusae* f.sp*. deltoidae* (*Mmd*), and *M. occidentalis* (*Mo*). Tukey's Honestly Significant Difference test was used to identify differences among genotype class means when a significant fixed effect was detected (shown in Table 2).

**Table S9**. Field surveys of fungal disease at Base de plein-air de Sainte-Foy. *Melampsora* spp*. i*ncidence was recorded as one of three damage classes: absent, <50% leaf area, and >50% leaf area. *Septoria* spp. leaf spot was recorded as presence or absence. Proportion of observed trees in each class is shown in brackets.
