# Supplementary material for: Fitness dynamics within a poplar hybrid zone: II. Impact of exotic sex on native poplars in an urban jungle
Source: Ecol Evol. 2014 Apr 19;4(10):1876–89. doi: 10.1002/ece3.1028 (PMC4063481; doi:10.1002/ece3.1028)
Supplement: Supplementary file 1 [file ece30004-1876-SD1.pdf]

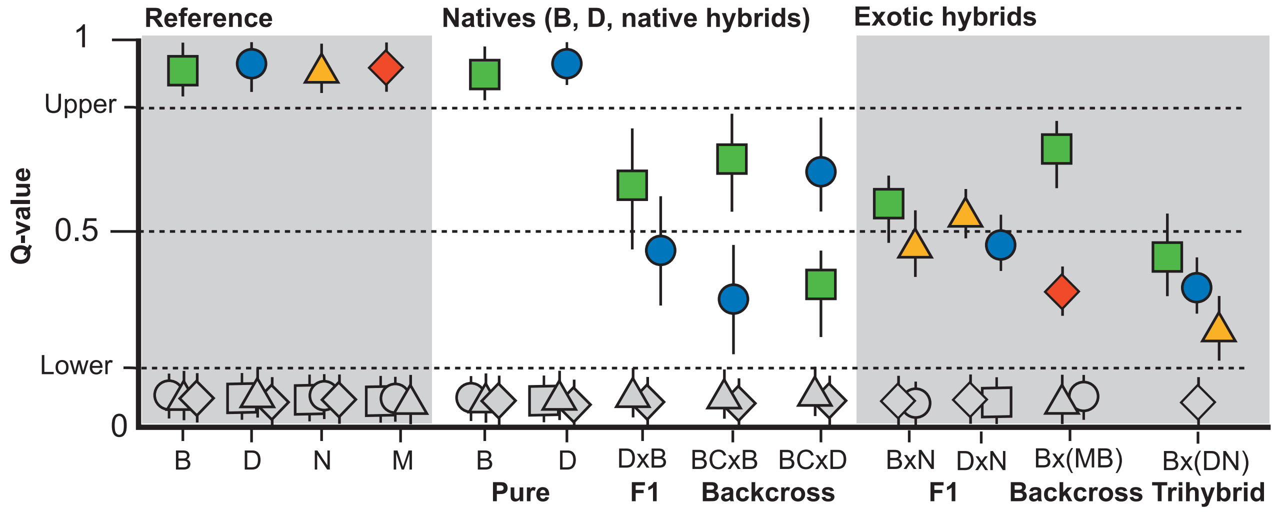

**Reference Population**	**Thresholds Upper**	**Lower**
P. balsamifera (■)	0.945	0.008
P. deltoides (●)	0.993	0.013
P. nigra (▲)	0.981	0.005
P. maximowiczii (◆)	0.988	0.047

Supplemental Figure 1

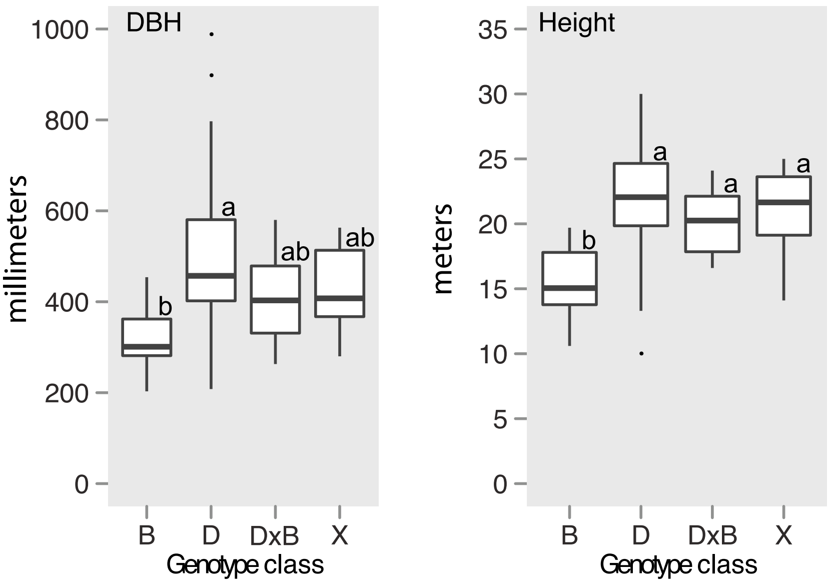

Supplemental Figure 2

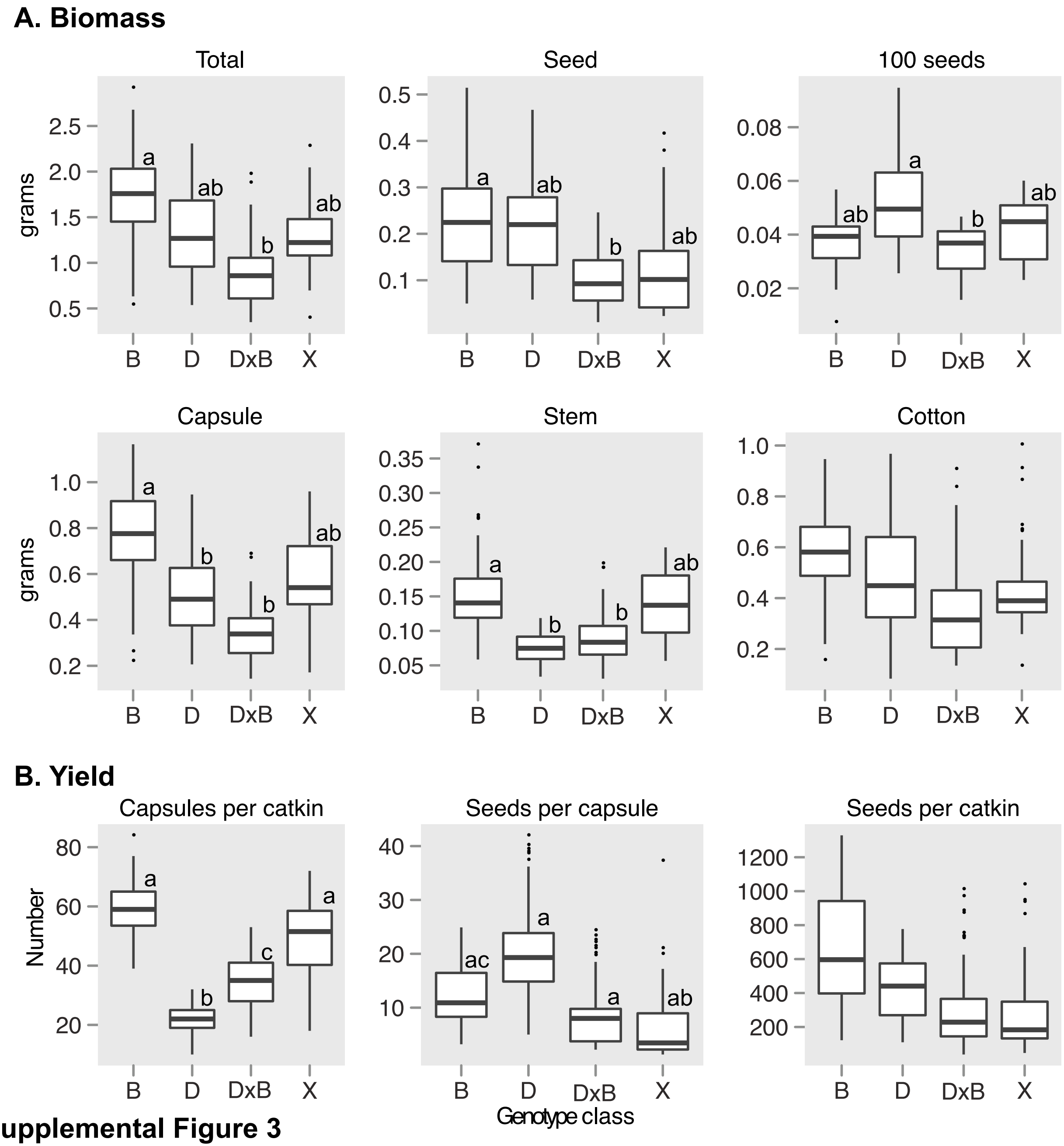

Supplemental Figure 3

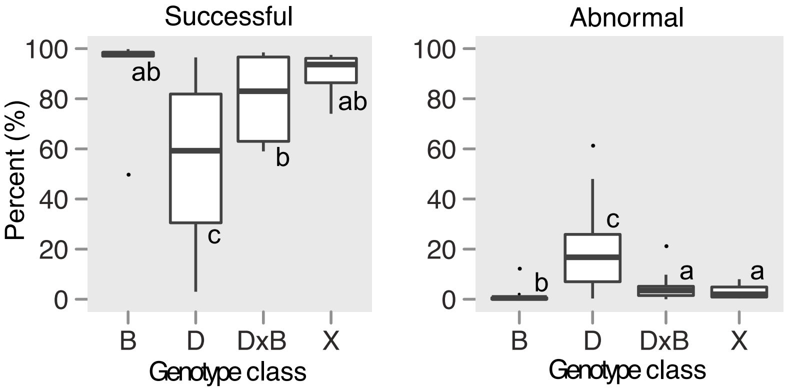

Supplemental Figure 4

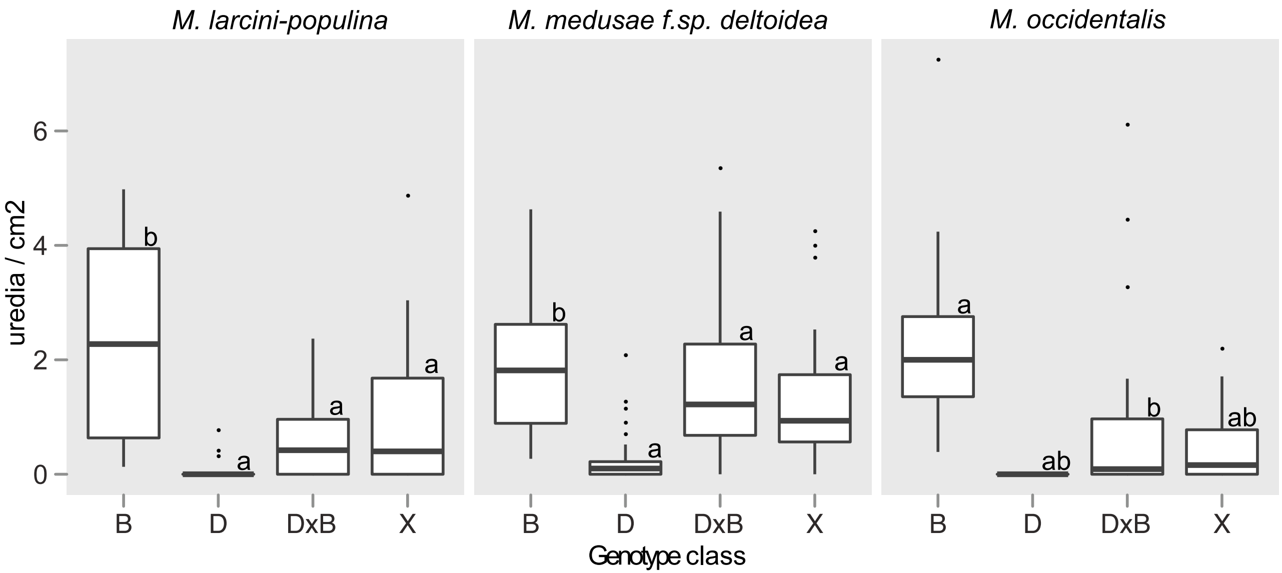

Supplemental Figure 5
