# Supplementary material for: Fitness dynamics within a poplar hybrid zone: II. Impact of exotic sex on native poplars in an urban jungle
Source: Ecol Evol. 2014 Apr 19;4(10):1876–89. doi: 10.1002/ece3.1028 (PMC4063481; doi:10.1002/ece3.1028)
Supplement: Supplementary file 2 [file ece30004-1876-SD2.doc]

**Table S1**. Diagnostic SNP markers for native and exotic poplar species in BPSF (Table adapted from Isabel *et al.* 2013). Major allele frequency is 1.0 unless indicated otherwise. Grey cells indicate the potential presence of a partial null allele. *Populus trichocarpa* *Nisqually-1* was used as a reference sequence

|  | **A Panel** | | | | | | | | | | | | | | | | |
| --- | --- | --- | --- | --- | --- | --- | --- | --- | --- | --- | --- | --- | --- | --- | --- | --- | --- |
| **Chromosome** | **VIII** | **X** | **V** | **IV** | **III** | **VI** | **XVI** | **XIII** | **V** | **XVII** | **X** | **IV** | **XI** | **IX** | **XVI** | **VIII** | **XIII** |
| **Gene Region** | **TB1** | **TB2** | **ATHB6** | **CO2** | **DIR2** | **CO4** | **VP** | **PHYA** | **WRKY3** | **ATHB3** | **TB2** | **PTAG** | **STM** | **CAD2** | **WRKY5** | **TB1** | **PHYA** |
| **SNP** | **004** | **005** | **006** | **007** | **008** | **009** | **016** | **019** | **021** | **024** | **025** | **031** | **032** | **034** | **038** | **040** | **041** |
| *Nisqually-1* (ref.) | T | T | C | C | T | A | G | T | T | T | T | T | T | T | C | G | C |
| *P. balsamifera* | • | • | • | • | • | • | • | • | • | • | • | • | • | • | • | A | C 0.99 |
| *P. deltoides* | C | C | T | G | G | T | T | C | C | G | • | • | • | • | • | A | T |
| *P. maximowiczii* | C | • | T | • | T 0.77 | T | • | • | • | • | • | • | • | • | • | A 0.90 | T |
| *P. nigra* | C | C | T | G | G | T | • | • | • | • | C 0.95 | C | C | A | T | A | T |

|  | **B Panel** | | | | | | | | | | | | | | | **F Panel** | | |
| --- | --- | --- | --- | --- | --- | --- | --- | --- | --- | --- | --- | --- | --- | --- | --- | --- | --- | --- |
| **Chromosome** | **I** | **III** | **VI** | **VII** | **X** | **XI** | **XII** | **XIV** | **II** | **III** | **IV** | **XV** | **VI** | **XIV** | **V** | **VIII** | **XI** | **XIX** |
| **Gene Region** | **CBF1** | **WRKY2** | **FBOX1** | **MAPK14** | **MAPK16** | **STM** | **MYB19** | **WRKY10** | **KN3** | **DIR2** | **NAC2** | **GA20OX** | **MKK2** | **CHS1** | **WRKY3** | **TB1** | **STM** | **KUNITZ2** |
| **SNP** | **001** | **003** | **010** | **011** | **013** | **014** | **015** | **017** | **021** | **022** | **023** | **027** | **028** | **029** | **035** | **001** | **002** | **003** |
| *Nisqually-1* (ref.) | A | A | T | G | A | C | T | T | T | T | A | A | A | C | C/T | A | C | C |
| *P. balsamifera* | C | G 0.99 | • | • | • | • | • | • | • | • | • | • | • | • | T 0.98 | • | • | • |
| *P. deltoides* | • | • | • | • | • | • | • | • | • | • | • | • | C | T | T | • | • | • |
| *P. maximowiczii* | • | G | G 0.50 | G 0.90 | A 0.82 | • | • | • | • | • | • | • | • | • | T | G | C 0.81 | T |
| *P. nigra* | • | • | • | • | • | • | • | • | C | A | G | T | C | C 0.51 | T | • | • | • |

**Data S1: R-code for analysis of fitness traits.**

See text file submitted to Dryad Data Repository (No. #######)

**Table S2**: Summary of half-sibling progeny from pure, hybrid, and exotic hybrid mothers. Putative fathers are inferred by haplotype substraction from maternal and seed genotypes (*P. balsamifera* = B, *P. deltoides* = D, *P. nigra* = N, *P. maximowiczii* =M; ? indicates that the father genotype could not be completely reconstructed with confidence).

| **Mother** | **Seed** |  |  |  |  |  | **Putative Father** | | | |  |  |  |  |  |  |  |  |  |
| --- | --- | --- | --- | --- | --- | --- | --- | --- | --- | --- | --- | --- | --- | --- | --- | --- | --- | --- | --- |
|  | n | B | D | native | exotic |  | B | D | N | DB | BN | MB | MBN | DBN | DN | B? | D? | BN? | DN? |
| ***P. balsamifera*** | |  |  |  |  |  |  |  |  |  |  |  |  |  |  |  |  |  |  |
| BPSF-004 | 48 | 47 | 0 | 0 | 1 |  | 47 |  |  |  |  |  | 1 |  |  |  |  |  |  |
| BPSF-053 | 48 | 46 | 0 | 0 | 2 |  | 46 |  |  |  |  | 2 |  |  |  |  |  |  |  |
| BPSF-072 | 47 | 45 | 0 | 1 | 1 |  | 45 |  |  | 1 |  |  |  | 1 |  |  |  |  |  |
| ***P. deltoides*** |  |  |  |  |  |  |  |  |  |  |  |  |  |  |  |  |  |  |  |
| BPSF-008 | 46 | 0 | 46 | 0 | 0 |  |  | 46 |  |  |  |  |  |  |  |  |  |  |  |
| BPSF-032 | 47 | 0 | 46 | 1 | 0 |  | 1 | 46 |  |  |  |  |  |  |  |  |  |  |  |
| BPSF-043 | 94 | 0 | 94 | 0 | 0 |  |  | 94 |  |  |  |  |  |  |  |  |  |  |  |
| BPSF-095 | 47 | 0 | 45 | 2 | 0 |  | 2 | 45 |  |  |  |  |  |  |  |  |  |  |  |
| **Native hybrids** | |  |  |  |  |  |  |  |  |  |  |  |  |  |  |  |  |  |  |
| BPSF-024 | 48 | 0 | 0 | 42(6*) | 0 |  | 17 | 30 |  | 1 |  |  |  |  |  |  |  |  |  |
| BPSF-034 | 47 | 0 | 0 | 42(1*) | 4 |  | 42 |  | 3 | 1 |  |  |  |  | 1 |  |  |  |  |
| BPSF-066 | 48 | 0 | 0 | 47(1*) | 0 |  | 18 | 28 |  | 2 |  |  |  |  |  |  |  |  |  |
| BPSF-071 | 47 | 0 | 0 | 46 | 1 |  | 45 |  |  | 1 |  | 1 |  |  |  |  |  |  |  |
| BPSF-077 | 47 | 0 | 0 | 47 | 0 |  | 46 |  |  | 1 |  |  |  |  |  |  |  |  |  |
| BPSF-082 | 47 | 0 | 0 | 47 | 0 |  | 40 | 2 |  | 5 |  |  |  |  |  |  |  |  |  |
| BPSF-088 | 48 | 0 | 0 | 43(3*) | 2 |  | 46 |  | 2 |  |  |  |  |  |  |  |  |  |  |
| **Exotic hybrids** | |  |  |  |  |  |  |  |  |  |  |  |  |  |  |  |  |  |  |
| BPSF-003 | 48 | 0 | 0 | 0 | 48 |  | 38 |  |  | 2 | 7 | 1 |  |  |  |  |  |  |  |
| BPSF-007 | 47 | 0 | 0 | 0 | 47 |  |  |  |  |  |  |  |  |  |  | 47 |  |  |  |
| BPSF-081 | 48 | 0 | 0 | 0 | 46(1*,1**) |  |  |  |  |  |  |  |  |  |  | 43 | 1 | 3 | 1 |
| BPSF-102 | 48 | 0 | 0 | 0 | 46(2***) |  | 43 |  |  | 3 | 1 |  | 1 |  |  |  |  |  |  |
|  |  |  |  |  |  |  |  |  |  |  |  |  |  |  |  |  |  |  |  |
| **Total** | 900 | 138 | 231 | 318  (11*) | 198  (1*,1**,2***) |  | 476 | 291 | 5 | 17 | 8 | 4 | 2 | 1 | 1 | 90 | 1 | 3 | 1 |

*Typed as pure but from native hybrid mother

**Typed as native hybrid but from exotic hybrid mother

***Typed as pure but from exotic mother

**Table S3**: Distribution of age classes estimated for reproductively mature *P. balsamifera*, *P. deltoides*, and native hybrids at BPSF.

|  |  | Estimated age (years) | | | |
| --- | --- | --- | --- | --- | --- |
|  | n | <20 | 20-40 | 40-60 | >60 |
| exotic hybrids | 14 | 0 | 13 | 1 | 0 |
| *P. balsamifera* | 14 | 5 | 9 | 0 | 0 |
| *P.deltoides* | 100 | 7 | 42 | 28 | 23 |
| native hybrids | 14 | 1 | 11 | 1 | 1 |

**Table S4:** Number of poplar trees measured for reproductive fitness and disease resistance traits: reproductive biomass, reproductive yield, seed viability, fungal disease susceptibility. Fungal disease was measure for three *Melampsora* species*: M. larcini-populina* (*Mlp*), *M. medusae f.sp. deltoidea* (*Mmd*), and *M. occidentalis* (*Mo*).Trees were grouped into four genotypic classes: pure *P. balsamifera* (B), pure *P. deltoides* (D), native hybrids (N), and exotic hybrids (X). Numbers in brackets indicate the number of trees sampled more than once.

|  |  | **2009** | |  |  |  | **2010** | |  |  |  | **2011** | |  |
| --- | --- | --- | --- | --- | --- | --- | --- | --- | --- | --- | --- | --- | --- | --- |
|  | B | D | N | X |  | B | D | N | X |  | B | D | N | X |
| Biomass | 2 | 3 | 3 | 2 |  | — | — | — | — |  | 5 (1) | 5 (2) | 3 (1) | 3 (2) |
| Yield | 2 | 3 | 3 | 2 |  | — | — | — | — |  | 5 (1) | 5 (2) | 3 (1) | 3 (2) |
| Viability | 3 | 17 | 7 | 4 |  | 4 (3) | 13 (11) | 5 (4) | 2 (1) |  | 5 (4) | 4 (3) | 3 (3) | 7 (1) |
| Fungus |  |  |  |  |  |  |  |  |  |  |  |  |  |  |
| *Mlp* | 3 | 8 | 7 | 6 |  | — | — | — | — |  | — | — | — | — |
| *Mmd* | 3 | 8 | 7 | 6 |  | — | — | — | — |  | — | — | — | — |
| *Mo* | 5 | 8 | 7 | 6 |  | — | — | — | — |  | — | — | — | — |

**Table S5**: Linear mixed effects model analysis summary for six biomass measurments taken on catkins from trees in *P. balsamifera* (B), *P. deltoides* (D), native hybrids (DxB), and exotic hybrids (X). 6A: Full data set; 6B: Partial data set of once sampled trees. Post-hoc tests (Tukey contrasts) were used to identify differences among genotype class means.

| **A. Full data set** |  |  | | | **Post-hoc** | |  |  |
| --- | --- | --- | --- | --- | --- | --- | --- | --- |
|  | factor | df | F | p | B | D | DxB | X |
| *total weight* |  |  |  |  |  |  |  |  |
|  | Intercept | 1,214 | 307.77268 | <.0001 |  |  |  |  |
|  | tree class | 3, 22 | 4.86416 | **0.0096**^†^ | a | ab | b | ab |
|  | year | 1,22 | 3.98373 | 0.0585 |  |  |  |  |
| *capsule weight* |  |  |  |  |  |  |  |  |
|  | Intercept | 1,214 | 316.8270 | <.0001 |  |  |  |  |
|  | tree class | 3,22 | 7.7449 | **0.0010** | a | b | b | ab |
|  | year | 1,22 | 1.1408 | 0.2971 |  |  |  |  |
| *cotton weight* |  |  |  |  |  |  |  |  |
|  | Intercept | 1,214 | 280.38763 | <.0001 |  |  |  |  |
|  | tree class | 3,22 | 1.92651 | 0.1548 |  |  |  |  |
|  | year | 1,22 | 10.66966 | **0.0035**^†^ |  |  |  |  |
| *hundred seed* |  |  |  |  |  |  |  |  |
| *weight* | Intercept | 1,214 | 380.9344 | <.0001 |  |  |  |  |
|  | tree class | 3,22 | 3.7735 | **0.0252** | ab | a | b | ab |
|  | year | 1,22 | 0.5186 | 0.4790 |  |  |  |  |
| *total seed* |  |  |  |  |  |  |  |  |
| *weight* | Intercept | 1,214 | 103.06856 | <.0001 |  |  |  |  |
|  | tree class | 3, 22 | 3.17972 | **0.0441**^†^ | a | ab | b | ab |
|  | year | 1,22 | 1.77314 | 0.1966 |  |  |  |  |
| *stem weight* |  |  |  |  |  |  |  |  |
|  | Intercept | 1,210 | 194.28520 | <.0001 |  |  |  |  |
|  | tree class | 3, 22 | 6.62524 | **0.0023** | a | b | b | ab |
|  | year | 1,22 | 0.00414 | 0.9493 |  |  |  |  |

| **B. Partial data set** | |  |  |  | **Post-hoc** | |  |  |
| --- | --- | --- | --- | --- | --- | --- | --- | --- |
|  | factor | df | F | p | B | D | DxB | X |
| *total weight* |  |  |  |  |  |  |  |  |
|  | Intercept | 1,125 | 116.63423 | <.0001 |  |  |  |  |
|  | tree class | 3,10 | 1.82622 | 0.2061 |  |  |  |  |
|  | year | 1,10 | 0.21772 | 0.6508 |  |  |  |  |
| *capsule weight* |  |  |  |  |  |  |  |  |
|  | Intercept | 1,125 | 153.29802 | <.0001 |  |  |  |  |
|  | tree class | 3,10 | 4.62077 | **0.0282** | a | ab | b | ab |
|  | year | 1,10 | 0.00033 | 0.9859 |  |  |  |  |
| *cotton weight* |  |  |  |  |  |  |  |  |
|  | Intercept | 1,125 | 92.29736 | <.0001 |  |  |  |  |
|  | tree class | 3,10 | 0.80954 | 0.5170 |  |  |  |  |
|  | year | 1,10 | 2.06619 | 0.1811 |  |  |  |  |
| *hundred seed* |  |  |  |  |  |  |  |  |
| *weight* | Intercept | 1,125 | 207.73026 | <.0001 |  |  |  |  |
|  | tree class | 3,10 | 5.60802 | **0.0162** | a | b | a | ab |
|  | year | 1,10 | 2.49988 | 0.1449 |  |  |  |  |
| *total seed* |  |  |  |  |  |  |  |  |
| *weight* | Intercept | 1,125 | 36.12203 | <.0001 |  |  |  |  |
|  | tree class | 3,10 | 1.80434 | 0.2100 |  |  |  |  |
|  | year | 1,10 | 0.04182 | 0.8421 |  |  |  |  |
| *stem weight* |  |  |  |  |  |  |  |  |
|  | Intercept | 1,125 | 239.13112 | <.0001 |  |  |  |  |
|  | tree class | 3,10 | 5.69210 | **0.0155** | ac | bc | c | a |
|  | year | 1,10 | 1.61368 | 0.2327 |  |  |  |  |

^†^Significance differs in partial (once-sampled tree) data set

**Table S6**: Linear mixed effects model analysis summary for the reproductive yield of catkins sampled *P. balsamifera* (B), *P. deltoides* (D), native hybrids (DxB), and exotic hybrids (X). 7A: Full data set; 7B: Partial data set. Post-hoc tests (Tukey contrasts) were used to identify differences among tree class means when a significant fixed effect was detected.

| **A: Full data set** |  | |  | | |  |  | | |  |  | | |  |  | | | Post-hoc | | | | | |
| --- | --- | --- | --- | --- | --- | --- | --- | --- | --- | --- | --- | --- | --- | --- | --- | --- | --- | --- | --- | --- | --- | --- | --- |
| response | predictors | | df | | | AIC | BIC | | | Log-Likelihood | χ^2^ | | | χ^2^ df | P | | | B | | D | | N | X |
| capsule yield | null* | | 2 | | | 311.06 | 317.90 | | | -153.53 |  | | |  |  | | |  | |  | |  |  |
|  | year | | 3 | | | 312.95 | 323.21 | | | -153.48 | 0.1068 | | | 1 | 0.7438 | | |  | |  | |  |  |
|  | tree class | | 5 | | | 279.78 | 296.88 | | | -134.89 | 37.1724 | | | 2 | **<0.0001** | | | a | | b | | c | a |
|  | year + tree class | | 6 | | | 280.18 | 300.70 | | | -134.09 | 1.6036 | | | 1 | 0.2054 | | |  | |  | |  |  |
|  |  | |  | | |  |  | | |  |  | | |  |  | | |  | |  | |  |  |
| number of seeds | null* | | 3 | | | 1122.1 | 1132.6 | | | -558.08 |  | | |  |  | | |  | |  | |  |  |
| per catkin | year | | 4 | | | 1121.2 | 1135.1 | | | -556.57 | 2.9620 | | | 1 | 0.0852 | | |  | |  | |  |  |
|  | tree class | | 6 | | | 1121.7 | 1142.6 | | | -554.85 | 3.4525 | | | 2 | 0.17795 | | |  | |  | |  |  |
|  | year + tree class | | 7 | | | 1118.5 | 1142.8 | | | -552.22 | 5.2466 | | | 1 | **0.02199**^†^ | | |  | |  | |  |  |
|  |  | |  | | |  |  | | |  |  | | |  |  | | |  | |  | |  |  |
| seeds per capsule | null* | | 3 | | | 975.50 | 985.76 | | | -484.75 |  | | |  |  | | |  | |  | |  |  |
|  | year | | 4 | | | 974.03 | 987.71 | | | -483.01 | 3.4702 | | | 1 | 0.0624 | | |  | |  | |  |  |
|  | tree class | | 6 | | | 969.28 | 989.81 | | | -478.64 | 8.7442 | | | 2 | **0.0126**^†^ | | | ac | | a | | c | bc |
|  | year + tree class | | 7 | | | 962.54 | 986.49 | | | -474.27 | 8.7398 | | | 1 | **0.0031** | | |  | |  | |  |  |
| ***B. Partial data set*** | |  | |  |  | | |  |  | | |  |  | | |  | **Post-hoc** | | | | | | |
| response | | predictors | | df | AIC | | | BIC | Log-Likelihood | | | *χ^2^* | *χ^2^* df | | | P | B | | D | | DxB | | X |
| capsule yield | | null* | | 3 | 159.74 | | | 165.47 | -77.868 | | |  |  | | |  |  | |  | |  | |  |
|  | | year | | 4 | 161.36 | | | 169.96 | -77.680 | | | 0.3747 | 1 | | | 0.5404 |  | |  | |  | |  |
|  | | tree class | | 6 | 141.77 | | | 156.11 | -65.887 | | | 23.5873 | 2 | | | **<0.0001** | a | | b | | c | | a |
|  | | year + tree class | | 7 | 142.44 | | | 159.65 | -65.222 | | | 1.3299 | 1 | | | 0.2488 |  | |  | |  | |  |
|  | |  | |  |  | | |  |  | | |  |  | | |  |  | |  | |  | |  |
| number of seeds | | null* | | 3 | 637.80 | | | 646.62 | -315.90 | | |  |  | | |  |  | |  | |  | |  |
|  | | year | | 4 | 638.88 | | | 650.65 | -315.44 | | | 0.9142 | 1 | | | 0.3390 |  | |  | |  | |  |
|  | | tree class | | 6 | 639.08 | | | 656.73 | -313.54 | | | 3.8010 | 2 | | | 0.1495 |  | |  | |  | |  |
|  | | year + tree class | | 7 | 639.48 | | | 660.07 | -312.74 | | | 1.6064 | 1 | | | 0.2050 |  | |  | |  | |  |
|  | |  | |  |  | | |  |  | | |  |  | | |  |  | |  | |  | |  |
| seeds per capsule | | null* | | 3 | 529.53 | | | 537.83 | -261.62 | | |  |  | | |  |  | |  | |  | |  |
|  | | year | | 4 | 527.74 | | | 539.21 | -259.87 | | | 3.4872 | 1 | | | 0.0618 |  | |  | |  | |  |
|  | | tree class | | 6 | 526.76 | | | 543.96 | -257.38 | | | 4.9861 | 2 | | | 0.0826 |  | |  | |  | |  |
|  | | year + tree class | | 7 | 520.13 | | | 540.21 | -253.07 | | | 8.6227 | 1 | | | **0.0032** |  | |  | |  | |  |

* random effects only ^†^ Significance differs between data sets

**Table S7**: General linear model analysis summary for viability of seed from P. balsamifera (B), P. deltoides (D), native hybrids (N), and exotic hybrids (X). Tree class and year were treated as fixed effects and thousand seed weight was included as a covariate. Tukey’s Honestly Significant Difference test was used to identify differences among tree class means when a significant effect was detected.

|  |  |  |  |  |  |  |  | Post-hoc | | | |
| --- | --- | --- | --- | --- | --- | --- | --- | --- | --- | --- | --- |
| test | factor | df | Deviance explained | Residual df | Residual deviance | P ( *χ*^2^ ) | F* | B | D | DxB | X |
| *germination 2009* | |  |  |  |  |  |  |  |  |  |  |
|  | null |  |  | 30 | 44.779 |  |  |  |  |  |  |
|  | TSW | 1 | 0.694 | 29 | 44.085 | 0.40479 | 0.6940 |  |  |  |  |
|  | tree class | 3 | 11.719 | 26 | 32.366 | **0.00841** | 3.9064 | ac | b | c | ac |
| *abnormal germination 2009* | |  |  |  |  |  |  |  |  |  |  |
|  | null |  |  | 30 | 77.726 |  |  |  |  |  |  |
|  | TSW | 1 | 2.601 | 29 | 75.125 | 0.1068 | 2.6009 |  |  |  |  |
|  | tree class | 3 | 41.921 | 26 | 33.204 | **<0.0001** | 13.9738 | a | b | c | c |

* F values are included for convenience. They should be interpreted with caution as the dispersion in these models is not constant.

**Table S8**: Linear mixed effects model analysis summary for infection severity (number of uredia) of poplar rust on P. balsamifera (B), P. deltoides (D), native hybrids (N), and exotic hybrids (X). Results are based on controlled inoculations of *Melampsora larici-populina* (*Mlp*), *M. medusae f.sp. deltoidae* (*Mmd*), and *M. occidentalis* (*Mo*). Tukey’s Honestly Significant Difference test was used to identify differences among tree class means when a significant fixed effect was detected.

|  |  |  |  |  |  |  |  | Post-hoc | | | |
| --- | --- | --- | --- | --- | --- | --- | --- | --- | --- | --- | --- |
| test | model | AIC | BIC | Log-Liklihood | *Χ^2^* | df | P | B | D | N | X |
| Mlp |  |  |  |  |  |  |  |  |  |  |  |
|  | 1 | 230.74 | 245.03 | -109.37 | 25.632 | 3 | **<0.001** | a | b | a | a |
|  | 2 | 250.37 | 257.52 | -122.19 |  |  |  |  |  |  |  |
| Mmd |  |  |  |  |  |  |  |  |  |  |  |
|  | 1 | 306.60 | 321.79 | -147.30 | 34.766 | 3 | **<0.001** | a | b | a | a |
|  | 2 | 335.36 | 342.96 | -164.68 |  |  |  |  |  |  |  |
| Mo |  |  |  |  |  |  |  |  |  |  |  |
|  | 1 | 230.73 | 246.54 | -109.36 | 29.693 | 3 | **0.001** | a | ab | b | ab |
|  | 2 | 254.42 | 262.33 | -124.21 |  |  |  |  |  |  |  |

model 1: number of uredia ~ tree class + random effects

model 2: number of uredia ~ random effects

random effects: tree, observation per tree

**Table S9**: Field surveys of fungal disease at BPSF. *Melampsora* spp. incidence was recorded as one of three damage classes: absent, <50% leaf area , and >50% leaf area. *Septoria* spp. leaf spot was recorded as presence or absence. Proportion of observed trees in each class is shown in brackets.

|  | *Melampsora* spp. | | | |  | *Septoria* spp. | | |
| --- | --- | --- | --- | --- | --- | --- | --- | --- |
| tree class | n | absent | <50% | >50% |  | n | absent | present |
| exotic hybrids | 8 | 4 (0.5) | 2 (0.25) | 2 (0.25) |  | 9 | 1 (0.11) | 8 (0.89) |
| *P. balsamifera* | 7 | 2 (0.29) | 3 (0.43) | 2 (0.29) |  | 8 | 0 | 8 (1.00) |
| native hybrids | 8 | 3 (0.38) | 3 (0.38) | 2 (0.25) |  | 11 | 0 | 11 (1.00) |
| *P. deltoides* | 64 | 30 (0.47) | 20 (0.31) | 14 (0.22) |  | 68 | 4 (0.06) | 64 (0.94) |
